# Interpersonal computational modelling of social synchrony in schizophrenia and beyond

**DOI:** 10.1093/psyrad/kkaf011

**Published:** 2025-05-05

**Authors:** Gwynnevere Suter, Emma Černis, Lei Zhang

**Affiliations:** School of Psychology, University of Birmingham, B15 2TT, UK; Institute for Mental Health, University of Birmingham, , B15 2TT, UK; Centre for Human Brain Health, University of Birmingham, B15 2TT, UK; School of Psychology, University of Birmingham, B15 2TT, UK; Institute for Mental Health, University of Birmingham, , B15 2TT, UK; School of Psychology, University of Birmingham, B15 2TT, UK; Institute for Mental Health, University of Birmingham, , B15 2TT, UK; Centre for Human Brain Health, University of Birmingham, B15 2TT, UK; Centre for Developmental Sciences, University of Birmingham, , B15 2TT, UK

**Keywords:** Interpersonal psychiatry, Computational psychiatry, Social coordination, Interpersonal computational modelling, Psychosis, Autism, Dissociation

## Introduction

Atypical social cognition and functioning constitute a significant proportion of the symptoms found in many psychiatric and neurodevelopmental conditions (Derntl & Habel, 2011; Rokita *et al*., 2018), such as autistic spectrum disorder (Cerullo *et al*., 2021), schizophrenia (Green *et al*., 2015), and social anxiety disorder (Alvi *et al*., 2022; Sohail & Zhang, 2024). Given that social coordination has existing associations with mental health (Macpherson *et al*., 2020; Dean *et al*., 2021; Macpherson & Miles, 2023) and a key role in everyday functioning, variation in coordination ability and strategy is a rich area of study for understanding these differences. Within this, much research has focused on interpersonal synchrony, the coordination and matching of behaviours and actions between two or more people (Yozevitch *et al*., 2023). Traditionally, however, attempts to study interpersonal synchrony have focused on analysing individuals rather than the complete dyad and the corresponding interaction (see Delaherche *et al*., 2012 and Fuchs *et al*., 2017 for details of measures). This can lead to missing crucial nuances in a dyad's dynamic, such as initial communication mismatch or deficits from one individual eliciting exaggerated compensatory behaviour in the other member, which masks the initial individual’s unusual communication (Olarewaju *et al*., 2023).

The emerging fields of interpersonal neuroscience and interpersonal psychiatry focus instead on analysing this interaction (Redcay & Schilbach, 2019; Dumas, 2022), and so access novel and arguably fundamental aspects of social coordination. This paradigmatic shift in focus facilitates, perhaps even necessitates, more naturalistic settings and tasks, in turn improving the general ecological validity of the field.

A recent perspective paper by Pan *et al*. (2023a) combined the principles of interpersonal psychiatry with computational psychiatry. Computational psychiatry aims to produce formal models of cognitive and neural processes and applies them to psychopathology to identify mechanisms of dysfunction (Friston *et al*., 2014; Huys *et al*., 2016; Zhang, 2023). The main contribution of their paper was a formal and population-general model of interpersonal dynamics in an isochronous finger tapping task, wherein two participants tap their fingers and need to achieve synchrony by listening to each other’s tapping (Heggli *et al*., 2019). Isochronous finger tapping tasks are common in interpersonal research and are used frequently in clinical populations (e.g. Konvalinka *et al*., 2010; Kawasaki *et al*., 2017; Wilquin *et al*., 2018). Pan and colleagues then applied this interpersonal computational model to the symptoms of schizophrenia and discussed potential outcomes.

**Figure 1:**
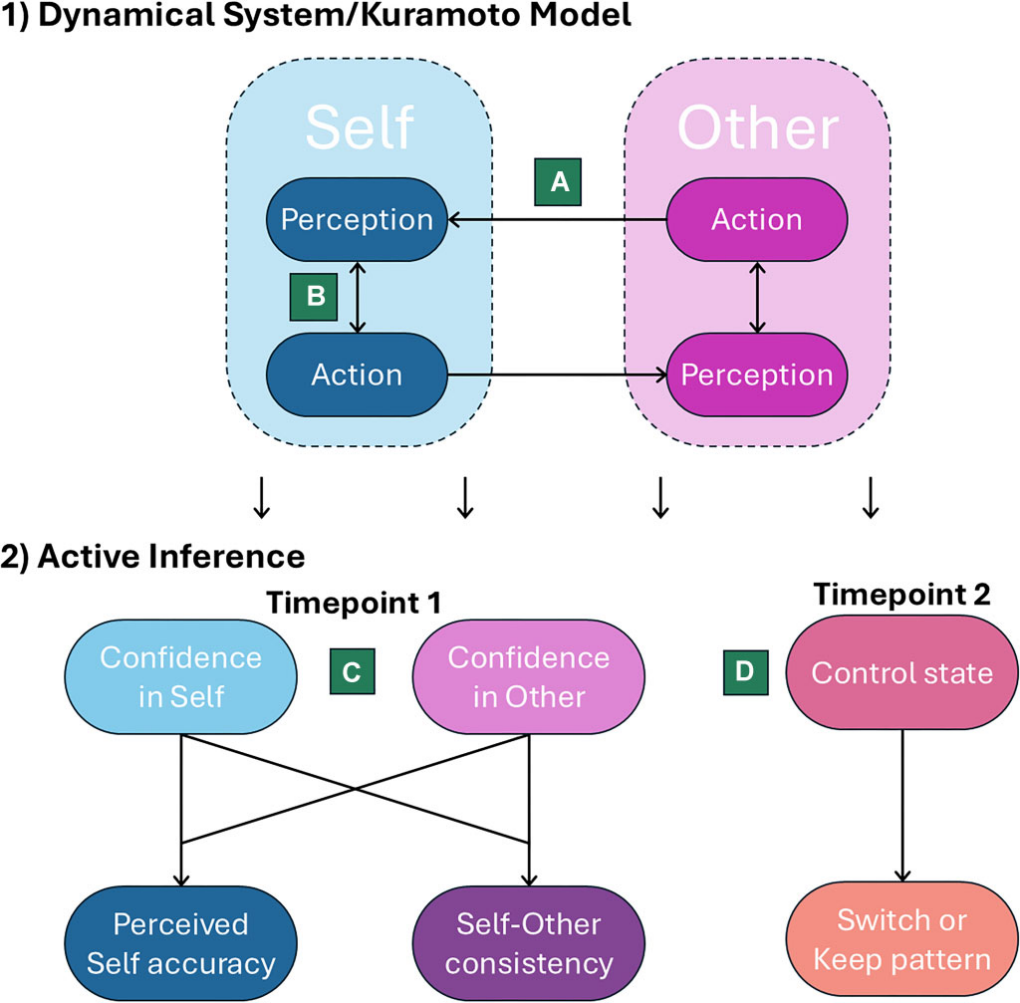
The interpersonal computational model of interpersonal synchrony during a coordinated tapping task. Adapted from Pan *et al*. (2023a). The figure illustrates the key processes suggested in the proposed interpersonal computational model. The first stage involves a Kuramoto model to establish weighting of within-agent and between-agent processes. The second stage invokes active inference to show how the confidence that the agent and/or the interaction partner are correct at Timepoint 1 determines the perceived accuracy of self and the self–other consistency, which then impacts the urge and finally decision to keep one's own pattern or switch to the partner's pattern at Timepoint 2. The letters in green boxes represent the four latent parameterized processes of the model: (**A**) desire for self–other consistency; (**B**) desire to be objectively accurate; (**C**) belief that one's own rhythm and/or their partner’s is correct; and (**D**) willingness to change to the partner’s rhythm.

Pan *et al*.’s (2023a) model in question has four core latent parameterized processes: the belief that the agent’s rhythm and/or the interaction partner’s rhythm is accurate; the desire to be objectively accurate; the desire to have the same rhythm as the partner; and willingness to change to the partner’s rhythm. These latent processes are structured in two stages (Fig. 1). The first stage recruits a dynamical systems model, the Kuramoto model, which estimates the weighting of within-agent and between-agent processes (Acebrón *et al*., 2005). This stage generates the initial parameters for the second stage. The second stage is based in an active inference framework and operates across the task's duration. At Timepoint 1, updating of beliefs, it estimates the confidence that the agent and the interaction partner respectively are correct, which determines the perceived accuracy of the agent and the consistency with the partner's rhythm. These beliefs impact the desire and decision to change to the partner's rhythm at Timepoint 2, action selection. The beliefs then continually update over time. With this multi-stage and multi-component model, the authors highlight that their paper could inspire potential developments in interpersonal computational psychiatry and that it might improve care for schizophrenia through improving subtyping for treatment and aiding drug development.

This current research highlight will discuss wider directions for their interpersonal computational model, beginning with other areas of schizophrenia research, before considering its application to different conditions such as autism spectrum disorder and dissociation and how applying the same model across multiple clinical contexts can facilitate direct comparison of disorders. This piece aims to illustrate the utility of the model and of an interpersonal computational approach.

## Within schizophrenia

The interpersonal computational model could test and refine theories of schizophrenia and/or psychosis. For example, the model is well suited to exploring the double bind theory of schizophrenia.

The double bind theory of schizophrenia posits a link between unusual ways of processing information and communicating with others, commonly associated with schizophrenia, and experiencing double bind interactions, particularly between a parent and child in early life (Bateson *et al*., 1956; Moskowitz & Montirosso, 2018). A double bind situation requires: multiple people, including the person in the bind; repeated interaction (if leading to psychosis); a threatening event from which the individual learns behavioural rules; a second threatening event conflicting with the rules learnt; and being unable to acknowledge the contradiction. For example, a parent chastises their child for being clingy, but when the child withdraws, they get punished for ignoring their parent and their protests are dismissed. This situation may result in difficulty interpreting others’ intentions and one’s own thoughts, causing typically inappropriate reactions and disorganized/confusing speech (Bateson *et al*., 1956; Moskowitz & Montirosso, 2018).

In computational terms, double binds may lead to people incorrectly inferring and weighting information from themselves and others. The model proposed by Pan *et al*. (2023a) could reveal potential cognitive changes occurring over a double bind situation. Monitoring participants’ latent model parameters in a coordinated finger tapping task before and after a double bind manipulation might reveal social coordination changes associated with experiencing this bind. We expect changes would be most noticeable in the amount of noise when weighting beliefs about self and other accuracy and in the desire to conform to the partner’s tapping rhythm (parameters C and D in Fig. 1), the direction of which would probably depend on the chronicity of the double binds and the dyad’s dynamic. This could further be combined with comparing estimated parameters from participants with and without psychosis to examine if double bind situations shift people towards a typically schizophrenic/psychotic manner of social coordination. This would both test the validity of the double bind theory and indicate whether and how social coordination strategies can be manipulated.

Other currently prominent theories of psychosis may not be as directly testable by this model. For example, Freeman *et al*.’s (2002) cognitive–behavioural model of persecutory delusions suggests that these delusions originate from the interaction between prior beliefs, personality and emotional traits, environmental factors, and specific cognitive biases such that unusual but significant experiences are interpreted as indicators of threat. These are then maintained by processes that reinforce the perception of threat: such as worry and the use of counter-productive safety-seeking behaviours (Freeman, 2016). Given this cognitive–behavioural model takes a holistic person-centred approach, rather than focusing on specific parameters of interpersonal interactions, the potential for communication between the models is scant. Therefore, it is unlikely the computational model could provide a significant contribution to this biopsychosocial cognitive–behavioural account of persecutory delusions.

Similarly, in the case of a predictive coding model of psychosis (Sterzer *et al*., 2018), the model’s predictions are too abstract to be potentially falsified by results from this interpersonal computational model. However, the interpersonal model may be applicable to the predictive coding account as a tool for exploring the computations underlying predictive coding during interpersonal interactions.

## Autism spectrum disorder

While initially discussed in the context of schizophrenia, the interpersonal computational model can be applied to studying other disorders. Autism spectrum disorder is commonly associated with abnormal social coordination (Cerullo *et al*., 2021), and so its research might benefit from using the model.

As suggested by Pan *et al*. (2023a), the model is designed to elucidate mechanistic differences in social coordination strategies, meaning it has the potential to better our understanding of the content of alterations in social coordination associated with autism.

The model might also offer insight into the dynamics underlying why people often show a preference towards interacting with others of the same neurotype (Chen *et al*., 2021) by, for example, correlating the strength and direction of preference with the model’s latent parameters and comparing the parameters across interactions within and between neurotypes.

Looking across conditions, we can use the interpersonal computational model to identify overlap (or lack thereof) in interpersonal synchrony strategies and processing across mental health conditions and neurodevelopmental disorders, which might in turn clarify the relation between conditions. For example, there is currently debate whether autism spectrum disorder and schizophrenia are diametrically opposed, particularly in the social domains (Crespi & Badcock, 2008), overlap significantly (King & Lord, 2011), or are related in other ways (Chisholm *et al*., 2015). One might inform this debate by comparing the model’s parameters in participants with autism and schizophrenia, which could indicate how much social cognition in these disorders overlap and differ, and identify specific processes of dis/similarity, such as different sensitivities to external input if they differ in their desire for self-other consistency (parameter A in Fig. 1).

These approaches could be combined with concurrent neuroimaging techniques such as hyperscanning with functional near-infrared spectroscopy (fNIRS), electroencephalography (EEG), or magnetoencephalography (MEG), to test if the brain-to-brain neural activity and connectivity underlying the model’s latent processes (e.g. Pan *et al*., 2023b) is disorder-specific or appears domain-general. This tightly reflects transdiagnostic approaches, such as the Research Domain Criteria (Insel *et al*., 2010) and The Hierarchical Taxonomy of Psychopathology (Kotov *et al*., 2017), which look at neurocognitive computational constructs that underlie a range of diagnostic presentations beyond categorical diagnoses.

## Dissociation

Dissociation is mentioned briefly by Pan *et al*. (2023a), where they define it as a possible consequence of imprecise priors and overweighted sensory evidence causing the false inference that something is absent. This definition does not lend dissociation and dissociative disorders to being studied with the proposed model. However, if we define dissociation as 'a disruption of and/or discontinuity in the normal integration of consciousness, memory, identity, emotion, perception, body representation, motor control, and behavior' (American Psychiatric Association, 2013, p. 291) (this quote refers originally to the core features of dissociative disorders), there is more scope for the model.

People experiencing dissociation may report that they do not feel in full control of their actions or that others around them feel unreal, though they understand that these feelings are untrue (Pienkos & Sass, 2022). As the interpersonal computational model is designed to estimate the confidence in self and others and the weighting of internal and external influences on decisions, it may be able to indicate whether these reports translate into actual behaviour. It is also well suited for further exploring the previous finding that low confidence in one’s abilities, self-efficacy, may be instrumental in developing and maintaining dissociation (Mahoney & Benight, 2019; Černis *et al*., 2022).

Double bind situations may be relevant for developing dissociation also, not just psychosis (Moskowitz & Montirosso, 2018), and so could bring insights similar to those discussed in the schizophrenia section.

Despite the theoretical relevance, there is currently very limited research on dissociation and social coordination, though initial work has indicated dissociation is associated with impaired interpersonal synchrony (Nieves, 2020). This means there is much room for studies on social coordination in dissociation generally, and that the interpersonal computational model could become a core part of the field.

Finally, dissociation is a widespread transdiagnostic experience (Lyssenko *et al*., 2018) and is found frequently in disorders associated with atypical social coordination such as autism spectrum disorder (Cerullo *et al*., 2021; Reuben & Parish, 2022), attention-deficit/hyperactivity disorder (Endo *et al*., 2006; Matsumoto & Imamura, 2007; Gvirts Problovski *et al*., 2021), and schizophrenia (O'Driscoll *et al*., 2014; Dean *et al*., 2021). Therefore, understanding the relation between social coordination and dissociation in isolation and in other disorders might tease apart contributions to an individual’s overall presentation and present specific targets for therapeutic intervention.

The interactions between social coordination, dissociation, and psychosis may be particularly useful to study given the historical (and current) confusion between dissociative and psychotic experiences and disorders (Middleton *et al*., 2018; Moskowitz & Heim, 2018) and given their significant overlap in risk factors (Irwin, 1999; Kelleher *et al*., 2013; Terock *et al*., 2016; Moskowitz & Montirosso, 2018; Stanton *et al*., 2020).

## Conclusion

Attention to interpersonal psychiatry and computational psychiatry is growing. Pan *et al*. (2023a) added to this by integrating the fields to produce an interpersonal computational model of social coordination and applying it to schizophrenia.

The present *Research Highlight* built on Pan *et al*. (2023a) by summarizing their model, before discussing its applications to other areas within schizophrenia, focusing on the double bind theory. Next, it considered how the model could contribute to research on autism spectrum disorder and the debate around the relation between autism and schizophrenia. The paper then finished with a suggestion of how interpersonal computational psychiatry could develop our understanding of dissociation and its transdiagnostic status.

Though it only discussed three groups of experiences/disorders, the model could be applied to many other issues in psychiatry. Of particular relevance are other conditions typically associated with atypical social functioning, such as social anxiety (Alvi *et al*., 2022), conduct disorder (Fairchild *et al*., 2019), and borderline personality disorder (Roepke *et al*., 2013). Future work should empirically test this task and model across various clinical conditions to begin establishing symptom- or disorder-specific parameter profiles. These profiles might ultimately be useable in predicting an individual’s disorder and/or subtype (Pan *et al*., 2023a).

While the model is best suited for furthering basic understanding of a disorder or trait, further work may establish how the model can be used in developing therapeutic or pharmacological interventions. For example, changes in model parameters over the course of treatment may be used as a proxy for real-world behavioural impacts and so be a useful and computationally meaningful outcome measure. In order to be incorporated into clinical practice, however, substantial empirical work would be required to demonstrate the model’s reliability in predicting disorders and/or subtypes and to streamline the implementation of the model for use in clinical settings.

By discussing a handful of potential applications of the proposed model, this research highlight aimed to explore the impact of Pan *et al*.’s (2023a) paper and to advocate the combination of interpersonal and computational approaches for studying neurodevelopmental and psychiatric experiences. It suggests the model and wider approach are indeed useful in a range of contexts, but that the model is not suited to all issues in psychiatry and further work is required particularly to prepare the model for clinical application.
